# Apgar scores correlate with survival rate at discharge in extremely preterm infants with gestational age of 25-27 weeks

**DOI:** 10.1590/1414-431X2022e12403

**Published:** 2022-12-02

**Authors:** Lili Lin, Guosheng Liu, Ying Li, Bijun Shi, Zhiwen Su, Chunhong Jia, Fan Wu

**Affiliations:** 1 Department of Neonatology, the First Affiliated Hospital of Jinan University, Guangzhou, Guangdong, China; 2 Department of Neonatology, the Third Affiliated Hospital of Guangzhou Medical University, Guangzhou, Guangdong, China; 3 Guangdong Provincial Key Laboratory of Major Obstetric Diseases, the Third Affiliated Hospital of Guangzhou Medical University, Guangzhou, Guangdong, China

**Keywords:** Apgar score, Extremely preterm infant, Survival, Outcome

## Abstract

Low Apgar score is associated with increased risk of death in preterm or full-term infants. However, the use of Apgar score to assess extremely preterm (EP) infants is controversial. In this study, we characterized the distribution of Apgar scores in EP infants with gestational age between 25 and 27 weeks, and investigated the association of Apgar score with survival rate at discharge by analyzing the clinical data of the EP infants discharged between January 2008 and December 2017 from 26 neonatal intensive care units in Guangdong Province, China. A total of 1567 infants with gestational age of 26.84±0.79 weeks and birth weight of 951±169 grams were involved in our study. The Apgar score increased with gestational age from 25 to 27 weeks and with time from birth from 1 to 10 min. The survival rate increased with a higher Apgar score, but no significant difference was found for 1-min Apgar score and the survival rate between infants with 25 or 26 weeks of gestation or 5-min Apgar score in infants with 25 weeks of gestation. The Apgar score is associated with survival of EP infants.

## Introduction

With advances in perinatology and neonatology, the survival rate of preterm infants, especially those with extremely low gestational age or extremely low birth weight, has greatly improved. However, neonatal asphyxia remains one of the leading causes for neonatal death and disability (1,2). Recently, a multicenter study in China also showed that complications related to preterm birth, intrapartum complications due to birth asphyxia, and congenital malformations were the top three causes of neonatal death (3). Effective assessment of adverse events at birth is of great significance for improving the poor prognosis of preterm infants. The Apgar score chart, established by Virginia Apgar in 1960, has become a universal tool for assessment of neonatal asphyxia (4). However, the American Academy of Pediatrics (AAP) and the American College of Obstetrics and Gynecology (ACOG) indicated that the score should not be used for any other purpose except for the assessment in the delivery room, because there were no consistent data on the significance of the Apgar score for preterm infants (5). In recent years, an increasing number of studies found that low Apgar scores, especially low Apgar score at 5 min, were associated with increased risk of death in preterm or full-term infants (6,7). Even in preterm infants born at 25 weeks of gestation, Apgar score can predict the mortality, especially very low Apgar score (0-3 points) (8). Currently, the Apgar score is one of the most used reference indicators by pediatricians to assess the prognosis of preterm infants (9).

Nevertheless, the use of Apgar score to assess extremely preterm (EP) infants (gestational age <28 weeks) is controversial. The low vitality, weak respiration, and reduced muscle tension exhibited by EP infants after birth may be due to organ immaturity rather than disease (10). Such factors have an adverse impact on making an accurate Apgar score. In this study, we aimed to characterize the distribution of Apgar scores in EP infants with gestational age between 25 and 27 weeks, and to investigate their association with survival rate at discharge by analyzing the clinical data of EP infants discharged between January 2008 and December 2017, from 26 neonatal intensive care units (NICUs) in Guangdong Province, China.

## Material and Methods

### Study population and data collection

This study was a secondary analysis of the data from the collaborative study group for EP infants as previously described (11). Given the high mortality rate of EP infants, only infants born between 25 and 27 weeks of gestation were eligible for this study. We collected data about maternal characteristics, pregnancy, and delivery as well as infant characteristics. A uniform standard of diagnosis and treatment were used in all participating NICUs during the study period. This study was approved by the Ethics Committee of the Third Affiliated Hospital of Guangzhou Medical University and written informed consent was obtained from the parents of all participants.

### Clinical definition

Survivors were defined as those infants who survived to the time of discharge. Gestational age was calculated from the date of the last menstrual period or determined by fetal ultrasound assessment. Neonatal respiratory distress syndrome (RDS) was diagnosed in preterm infants with the onset of respiratory distress shortly after birth, which may or may not have been combined with a compatible chest radiograph (12). Necrotizing enterocolitis (NEC) was assessed in the infants who survived more than two days after birth, and was diagnosed and graded based on the revised Bell's stage (13). Retinopathy of prematurity (ROP) was checked by indirect ophthalmoscope or ret-camera, and the graded standard were defined by the ROP international classification (14). Intraventricular hemorrhage (IVH) and periventricular leukomalacia (PVL) were diagnosed by cranial ultrasonography or magnetic resonance imaging (MRI). The Papile grading system was used to grade the severity of IVH, and PVL was defined as the degeneration of white matter adjacent to the cerebral ventricles following cerebral hypoxia or brain ischemia (15). Bronchopulmonary dysplasia (BPD) was defined as continuous oxygen dependency at 28 days of age and only assessed in the infants who survived more than 28 days (16).

### Statistical analysis

All statistical analyses were performed using SPSS 23.0 for Windows (IBM, USA). Continuous variables are reported as mean±SD. Categorical variables are reported as odds ratios and 95% confidence intervals (CI), and the chi-squared test was used for analysis. P<0.05 was considered statistically significant.

## Results

### Demographics of the EP infants and the mothers

A total of 2057 EP infants were discharged from the 26 participating NICUs during 2008-2017. There were 490 infants excluded, including 131 infants with a gestational age less than 25 weeks and 359 infants without Apgar scores at 10 min. Ultimately, a total of 1567 infants with mean gestational age of 26.84±0.79 weeks and mean birth weight of 951±169 grams were analyzed. There were 950 males (60.6%), 609 twins or multiple gestation (38.9%), and 330 (21.1%) infants delivered by cesarean section (Table 1).

**Table 1:** Population characteristics of the included infants and mothers.

Infants' characteristics	25 weeks (n=221)	26 weeks (n=450)	27 weeks (n=896)	Total (n=1567)
Birth weight (g), mean±SD	799±134	912±127	1008±164	951±169
Gender				
Male, n (%)	139 (62.9)	267 (59.3)	544 (60.7)	950 (60.6)
Female, n (%)	82 (37.1)	183 (40.7)	352 (39.3)	617 (39.4)
Intrauterine distress, n (%)	3 (1.4)	6 (1.3)	30 (3.3)	39 (2.5)
Admission to general hospital, n (%)	116 (52.5)	249 (55.3)	520 (58.0)	885 (56.5)
Diagnosed RDS, n (%)				
Yes	199 (90.0)	394 (87.6)	801 (89.4)	1394 (89.0)
No	22 (10.0)	56 (12.4)	95 (10.6)	173 (11.0)
BPD at 28 days, n (%)				
Yes	61 (82.4)	187 (84.2)	400 (69.8)	648 (74.6)
No	13 (17.6)	35 (15.8)	173 (30.2)	221 (25.4)
NEC, n (%)				
Yes	7 (5.3)	38 (11.8)	90 (12.6)	135 (11.6)
No	124 (94.7)	284 (88.2)	625 (87.4)	1033 (88.4)
ROP, n (%)				
Yes	45 (56.3)	129 (58.6)	198 (36.3)	372 (44.0)
No	35 (43.7)	91 (41.4)	347 (63.7)	473 (56.0)
IVH, n (%)				
Yes	68 (48.2)	118 (38.8)	193 (28.1)	379 (33.5)
No	73 (51.8)	186 (61.2)	493 (71.9)	752 (66.5)
PVL, n (%)				
Yes	9 (6.4)	22 (7.2)	43 (6.3)	74 (6.5)
No	132 (93.6)	282 (92.8)	643 (93.7)	1057 (93.5)
Survived at discharge, n (%)	69 (31.2)	201 (44.7)	540 (60.3)	810 (51.7)
Mothers' characteristics				
Twin/multiple gestation, n (%)	96 (43.4)	198 (44.0)	315 (35.1)	609 (38.9)
Cesarean section, n (%)	29 (13.1)	71 (15.8)	230 (25.7)	330 (21.1)
Antenatal steroids, n (%)	95 (43.0)	207 (46.0)	470 (52.5)	772 (49.3)
Premature rupture of membranes, n (%)	37 (16.7)	108 (24.0)	241 (26.9)	386 (24.6)
Placental abruption, n (%)	5 (2.3)	17 (3.8)	38 (4.2)	60 (3.8)
Placenta previa, n (%)	8 (3.6)	16 (3.6)	51 (5.7)	75 (4.8)
Abnormal amniotic fluid, n (%)	11 (5.0)	35 (7.8)	84 (9.4)	130 (8.3)
Antepartum infection, n (%)	16 (7.2)	40 (8.9)	68 (7.6)	124 (8.0)
Gestational diabetes mellitus, n (%)	10 (4.5)	45 (10.0)	103 (11.5)	158 (10.1)
Hypertensive disorders in pregnancy, n (%)	18 (8.1)	28 (6.2)	79 (8.9)	125 (8.0)
Cervical insufficiency, n (%)	12 (5.4)	21 (4.7)	26 (2.9)	59 (3.8)

RDS: respiratory distress syndrome; BPD: bronchopulmonary dysplasia; NEC: necrotizing enterocolitis; ROP: retinopathy of prematurity; IVH: intraventricular hemorrhage; PVL: periventricular leukomalacia.

### Distribution of Apgar scores in EP infants

Overall, the Apgar score increased between gestational ages of 25 and 27 weeks and between the time points of 1 and 10 min after birth. At 1 min, 68.3% of the infants with 25 weeks of gestation had Apgar scores less than 8 points, but in infants with 26 and 27 weeks of gestation, it was 52.7 and 44.3%, respectively. At 5 min, the proportion of Apgar scores ranging from 8 to10 points in the infants with 25, 26, and 27 weeks of gestation were 74.6, 78.7, and 81.2%, while the corresponding percentages at 10 min were 81.9, 85.6, and 87.3%, respectively (Supplementary Table S1).

### Apgar score and survival rate at discharge

The infants were divided into three groups according to the Apgar score into 0 to 3 points group, 4 to 7 points group, and 8 to 10 points group. As a whole, the survival rate increased with higher Apgar scores at all three time points, but not in all gestational age subgroups. No significant difference was found in 1-min Apgar score and the survival rate between the infants with 25 and 26 weeks of gestation, or between 1- and 5-min Apgar score in the infants with 25 weeks of gestation (P>0.05, Table 2).

**Table 2:** Survival rate at discharge according to Apgar score and gestational age.

	0-3 points		4-7 points		8-10 points		χ^2^	P
	n	Survivor, n (%)	n	Survivor, n (%)	n	Survivor, n (%)		
At 1 min								
25 W	40	13 (32.5)	111	32 (28.8)	70	24 (34.3)	0.632	0.729
26 W	65	20 (30.8)	172	82 (47.7)	213	99 (46.5)	5.992	0.050
27 W	98	41 (41.8)	299	165 (55.2)	499	334 (66.9)	26.390	0.000
Total	203	74 (36.5)	582	279 (47.9)	782	457 (58.4)	36.421	0.000
At 5 min								
25 W	10	2 (20)	46	12 (26.1)	165	55 (33.3)	1.493	0.473
26 W	16	2 (12.5)	80	24 (30)	354	175 (49.4)	16.918	0.000
27 W	20	3 (15)	149	75 (50.3)	727	462 (63.5)	26.521	0.000
Total	46	7 (15.2)	275	111 (40.4)	1246	692 (55.5)	46.020	0.000
At 10 min								
25 W	4	0 (0)	36	6 (16.7)	181	63 (34.8)	5.984*	0.014
26 W	6	0 (0)	59	17 (28.8)	385	184 (47.8)	5.528*	0.019
27 W	11	2 (18.2)	84	36 (42.9)	801	502 (62.7)	27.703	0.000
Total	21	2 (9.5)	179	59 (33.0)	1367	749 (54.8)	45.362	0.000

* Comparisons were made between groups 4-7 points and 8-10 points. W: weeks.

## Discussion

Many perinatal factors, such as maternal comorbidities, fetal status, and treatment during delivery, can affect the Apgar score and the outcome of the newborn. In this study, we focused on the correlation between Apgar scores and survival rates at discharge in EP infants with 25-27 weeks of gestation. The results showed different correlations between Apgar score and survival rate at discharge. In the infants with gestational age of 25 weeks, the survival rate at discharge increased with the higher Apgar scores only at 10 min, while in the infants with gestational age of 26 weeks, a similar result was found at 5 and 10 min. However, in the infants with gestational age of 27 weeks, survival rates were higher at 1, 5, and 10 min.

The value of Apgar scores for assessing the outcomes of EP infants has been questioned for many years, since the Apgar score was established 60 years ago when the limit of fetal viability was well above 25-27 weeks. The Apgar may have a prognostic value, but with limitations. The distribution of Apgar scores is associated with gestational age. The infants with lower gestational age have a higher proportion of low Apgar scores (17). In clinical practice, the Apgar score of an infant during resuscitation is not equivalent to that of a spontaneously breathing infant. In addition, the Apgar score may be affected by many factors besides gestational age, such as low birth weight, prolonged second stage of labor, previous induced abortion, preeclampsia, drugs used for anesthesia or analgesia, and the impression of the evaluators (9,10,18,19). As a result, the association of Apgar scores and the outcomes of EP infants seems to be weaker when compared to the full-term infants. Several studies demonstrated a poor correlation between Apgar scores and survival rates of very low birth weight or extremely low gestational age infants (20,21). However, our study revealed that the Apgar score was partly correlated with survival rate at discharge in EP infants with a gestational age of 25-27 weeks.

More in detail, our study showed that the infants with gestational age of 27 weeks had the highest proportion of normal (8-10 points) or intermediate Apgar score (4-7 points) among all EP infants, and the survival rate at discharge increased with higher Apgar scores at all the three time points. This was partly consistent with the studies of Lee et al. (17) and Cnattingius et al. (22)]. In the former study, Lee et al. (17) found that infants with greater gestational age had higher Apgar scores, and there was a positive correlation between high 5-min Apgar score and the survival rate in EP infants. In the latter research, Cnattingius et al. (22) noted that the Apgar scores at 5 and 10 min provided prognostic information about neonatal survival among preterm infants with gestational ages ranging from 22-36 weeks.

The severe immaturity of EP infants with a gestational age of 25 weeks can be an important factor that affects the Apgar score. In our study, the proportion of Apgar scores less than 8 points in infants with gestational age of 25 weeks was 68.3, 25.4, and 18.1% at 1, 5, and 10 min, respectively. Previous studies had demonstrated the impact of immaturity on Apgar scores, and the limited prognostic accuracy of Apgar scores for EP infants. The 1-min Apgar score might be correlated to the survival of neonates with 23-26 weeks of gestation when it is very low (0-1 point) (20,23). Similarly, we failed to find a significant correlation between the survival rate in infants with gestational age of 25 weeks and the Apgar score at 1 and 5 min. Differently, our study showed that a higher Apgar score at 10 min was correlated with increased survival rate at discharge in the infants with 25 weeks of gestation. This indicated that 10-min Apgar score can be used for predicting the survival of infants with 25 weeks of gestation. Combined with the results found in the infants with 26 and 27 weeks of gestation, we can speculate that the influence of immaturity on Apgar score weakens with increased gestational age and longer time after birth. Thus, the importance of assessing the Apgar score up to 10 min in EP infants should be emphasized, though physicians have always considered that it is not an indication for neonatal resuscitation. Effective resuscitation can increase Apgar score. However, prenatal care registration and consultation is important for EP infants and can help physicians make proper preparation for resuscitation.

In conclusion, although the application of the Apgar score in preterm infants has many limitations, our study still suggested that the Apgar score was partly correlated to the survival rate of EP infants at discharge. The correlation varied depending on the gestational age and the Apgar score at different time points. It can partly help pediatricians to rapidly assess the prognosis of these infants.

However, this study also has some limitations. First, the study population included only EP infants discharged from the NICUs rather than the entire birth population. Second, the Apgar score was subjective, although the 26 centers included in the study were from the same region and worked with similar protocols. Third, the gestational age might be inaccurate, since not all patients were assessed through ultrasound in the first trimester, which was the method used as a reference for gestational age. Further studies are needed.

## Supplementary Material

Click to view [pdf].
